# Supplementary material for: MethPhaser: methylation-based long-read haplotype phasing of human genomes
Source: Nat Commun. 2024 Jun 22;15:5327. doi: 10.1038/s41467-024-49588-0 (PMC11193733; doi:10.1038/s41467-024-49588-0)
Supplement: Supplementary file 1 — Supplementary Information [file 41467_2024_49588_MOESM1_ESM.pdf]

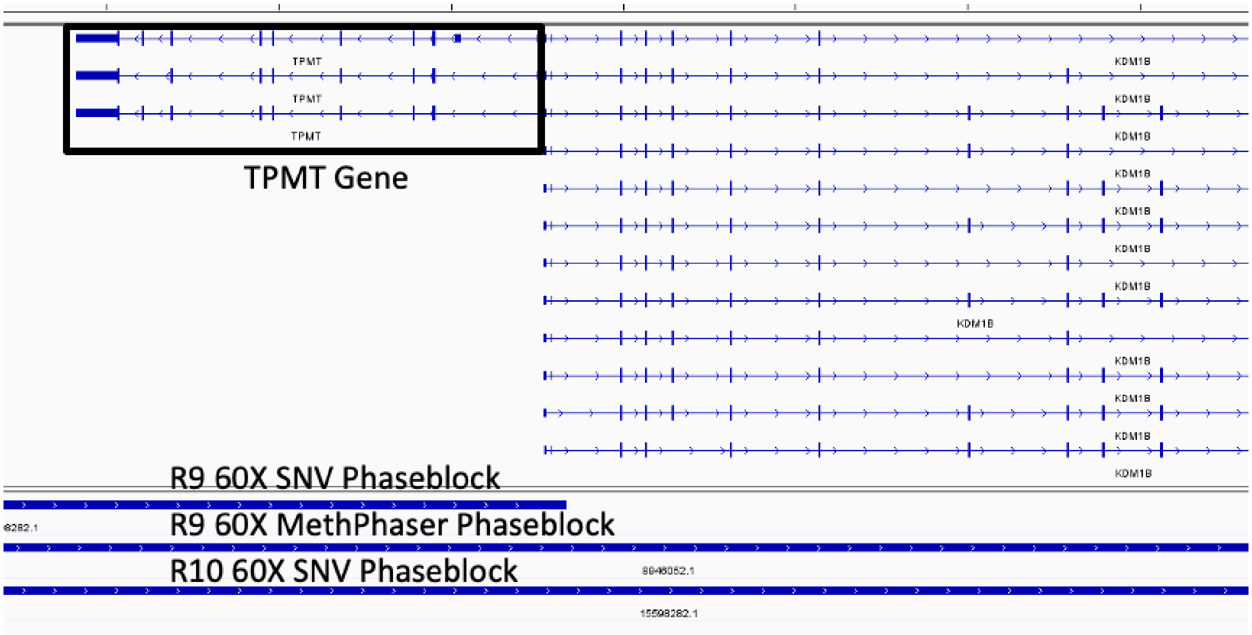

Supplementary Figure 1. Improved phasing around the Thiopurine methyltransferase (TPMT) gene (associated with encoding the enzyme metabolizing thiopurine drugs) as an important example. An IGV plot showing a TPMT example. With R9 60X reads, the SNV-based method cannot fully phase the TPMT region, while MethPhaser is able to phase the TPMT gene. The R10 60X SNV-based method can also phase the TPMT gene.

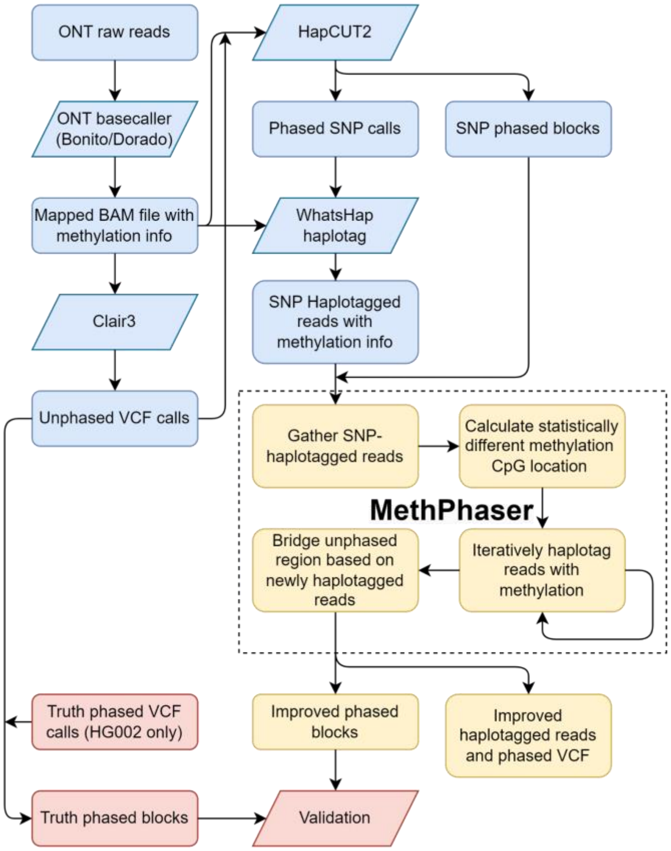

Supplementary Figure 2. Flowchart of MethPhaser Benchmarking Process. Blue blocks represent the traditional SNV-based phasing process, which is the SNV phasing that was used in this work. The yellow blocks, MethPhaser, were directly attached to the traditional SNV-based phasing pipeline for improvement. Red blocks represent the validation process we took in this paper with HG002;

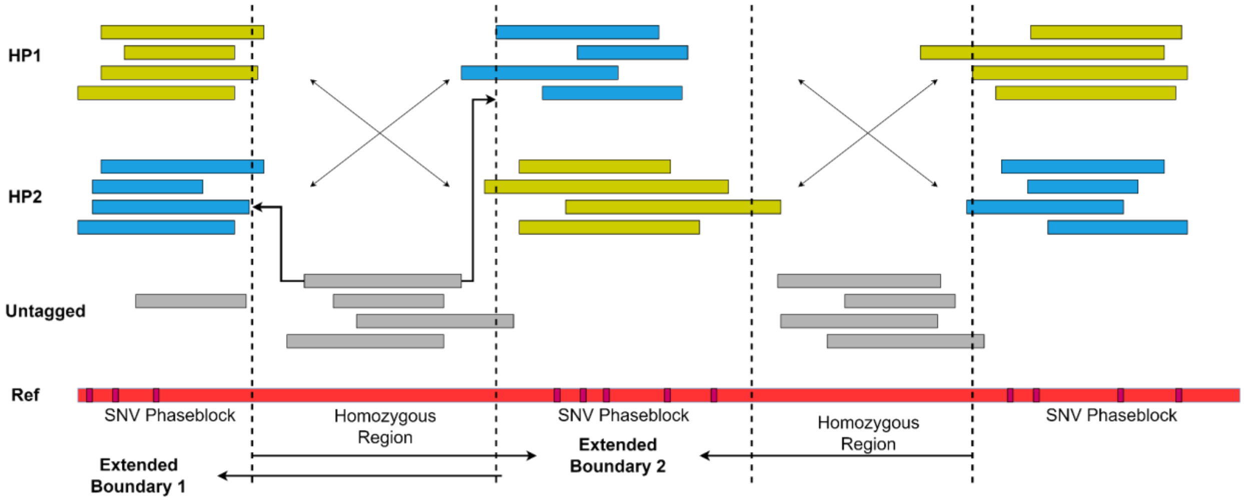

Supplementary Figure 3: The schematic illustration of MethPhaser's block connection process. We defined several extended boundaries for our classifier for each phaseblock (see method). And we now can start to infer the relationship between the neighbor phaseblocks. Each phaseblock has its own assignments for untagged reads, and the reads in unphased gaps can be assigned by both classified built from phaseblocks based on our definition of extended boundary. So for each previous untagged read that can be tagged by the classifiers we built from the previous SNV phaseblock and the next SNV phaseblock, we can check if those classifiers are outputting the same haplotype assignment or opposite haplotype assignment. If multiple reads support the opposite haplotype assignment, we can infer that the two SNV phaseblocks are having switched haplotype assignment relationship, and further close the gap. Or if the reads support the same haplotype assignment, we can infer that the two SNV phaseblocks are having the same haplotype assignment.

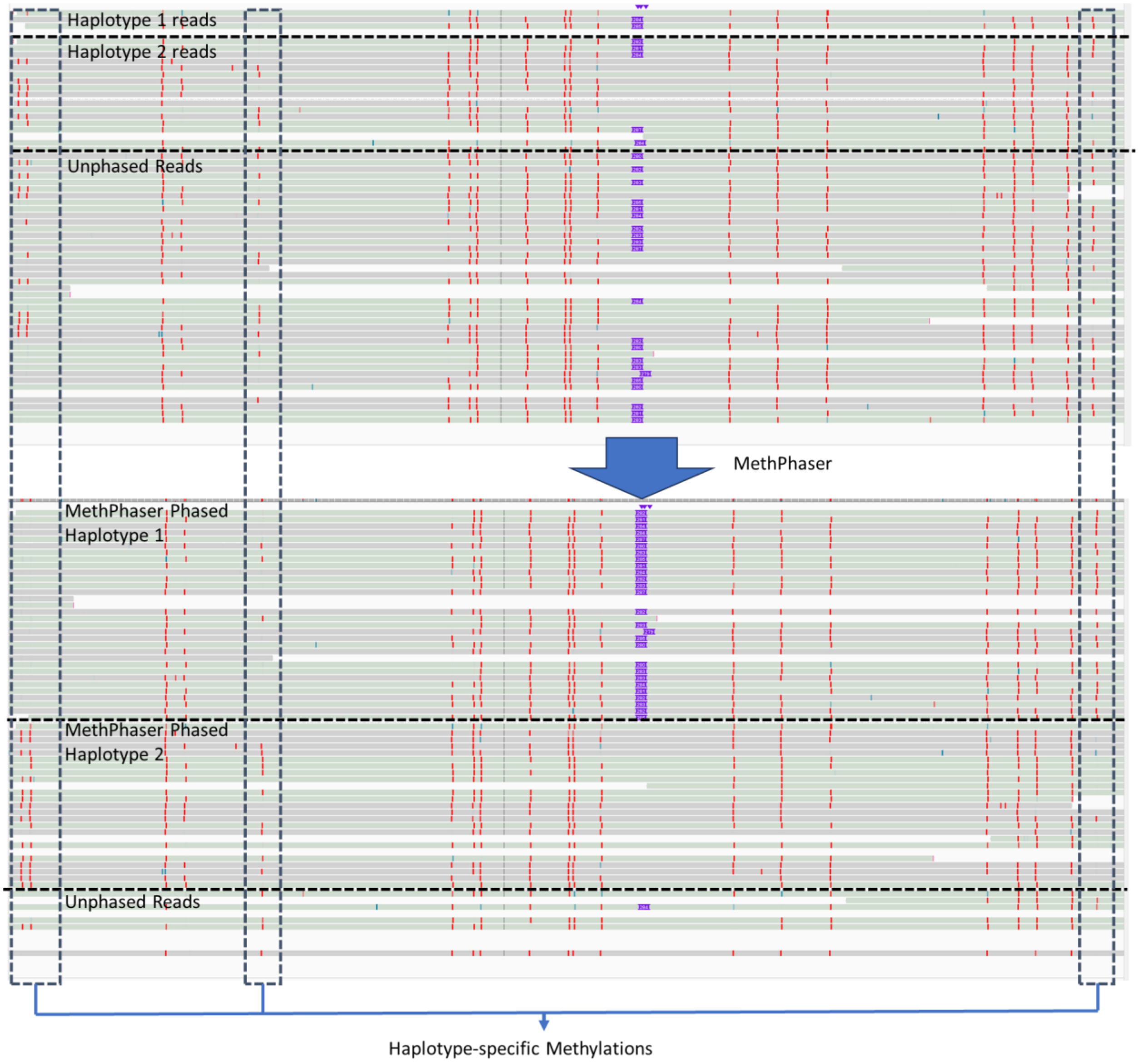

Supplementary Figure 4: MethPhaser phased an haplotype-specific insertion (chr6:30,893,462-30,894,563). This is a sub region of the HLA regions in Figure 4 and in which MethPhaser is able to utilize haplotype-specific methylations (marked by purple squares) to perform read phasing. The insertions are all phased into a single haplotype, except one read remain unphased, which highlights MethPhaser's ability of improving phasing.

Name	Version	Purpose	Command
minimap2	v2.24	raw read mapping to reference and keep MM, ML tags	minimap2 -t THREADS -y MM,ML -ax map-ont GCA_000001405.15_GRCh38_no_alt_analysis_set.fna
Clair3	v0.1-r12	call SNVs, HG002/pangenome R9 read SNV calling	run_clair3.sh --bam_fn=BAM --ref_fn=REF --output=OUTPUT_DIR --threads=THREADS --platform=PLATFORM --model_path=r941_prom_sup_g5014
Clair3	v0.1-r12	call SNVs, HG002 R10 read SNV calling	run_clair3.sh --bam_fn=BAM --ref_fn=REF --output=OUTPUT_DIR --threads=THREADS --platform=PLATFORM --model_path=r1041_e82_400bps_sup_g615
HapCUT2	v1.3.1	Phase SNVs	extractHAIRS --bam BAM --vcf Clair3_called_vcf --ref GCA_000001405.15_GRCh38_no_alt_analysis_set.fna --ont 1 --out FRAGMENT
HapCUT2	v1.3.1	Phase SNVs	hapcut2 --outvcf 1 --f FRAGMENT --VCF Clair3_called_vcf --o OUTPUT
WhatsHap	v1.6	get phaseblocks from SNV phased result	whatshap stats --gtf=GTF Phased_VCF
WhatsHap	v1.6	Haplotag reads	whatshap haplotag -o haplotagged_BAM --reference GCA_000001405.15_GRCh38_no_alt_analysis_set.fna --ignore-read-groups phased_VCF mapped_BAM
WhatsHap	v1.6	Calculate switch error and flip error	whatshap compare --ignore-sample-name --tsv-pairwise eval.tsv truth.vcf MethPhaser.vcf
SAMtools	v1.16.1	only keep primary alignments	samtools view -bF 2304 input.bam > primary.bam
MethPhaser	V0.0.1	Retrive blocks relationships based on SNV phased reads and methyaltion signals	meth_phaser_parallel -b haplotagged_BAM .bam -r GCA_000001405.15_GRCh38_no_alt_analysis_set.fna -g GTF -vc Phased_VCF -o WORK/
MethPhaser	V0.0.1	Alter BAM and VCF files	meth_phaser_post_processing -ib haplotagged_BAM -if WORK/ -ov OUTPUT.vcf -ob OUTPUT/ -vc Phased_VCF

Supplementary Table 1: The parameters of running the tools for benchmarking
