# Supplementary material for: MethPhaser: methylation-based long-read haplotype phasing of human genomes
Source: Nat Commun. 2024 Jun 22;15:5327. doi: 10.1038/s41467-024-49588-0 (PMC11193733; doi:10.1038/s41467-024-49588-0)
Supplement: Supplementary file 2 — Reporting Summary [file 41467_2024_49588_MOESM2_ESM.pdf]

Corresponding author(s): Fritz Sedlazeck

Last updated by author(s): May 20, 2024

Reporting Summary

Nature Portfolio wishes to improve the reproducibility of the work that we publish. This form provides structure for consistency and transparency in reporting. For further information on Nature Portfolio policies, see our Editorial Policies and the Editorial Policy Checklist.

Statistics

For all statistical analyses, confirm that the following items are present in the figure legend, table legend, main text, or Methods section.

n/a	Confirmed	
☐	☒	The exact sample size (*n*) for each experimental group/condition, given as a discrete number and unit of measurement
☐	☒	A statement on whether measurements were taken from distinct samples or whether the same sample was measured repeatedly
☐	☒	The statistical test(s) used AND whether they are one- or two-sided<br>*Only common tests should be described solely by name; describe more complex techniques in the Methods section.*
☒	☐	A description of all covariates tested
☒	☐	A description of any assumptions or corrections, such as tests of normality and adjustment for multiple comparisons
☐	☒	A full description of the statistical parameters including central tendency (e.g. means) or other basic estimates (e.g. regression coefficient) AND variation (e.g. standard deviation) or associated estimates of uncertainty (e.g. confidence intervals)
☐	☒	For null hypothesis testing, the test statistic (e.g. *F*, *t*, *r*) with confidence intervals, effect sizes, degrees of freedom and *P* value noted<br>*Give P values as exact values whenever suitable.*
☒	☐	For Bayesian analysis, information on the choice of priors and Markov chain Monte Carlo settings
☒	☐	For hierarchical and complex designs, identification of the appropriate level for tests and full reporting of outcomes
☒	☐	Estimates of effect sizes (e.g. Cohen's *d*, Pearson's *r*), indicating how they were calculated

Our web collection on statistics for biologists contains articles on many of the points above.

Software and code

Policy information about availability of computer code

Data collection	The public available data has been provided with download links in the manuscript. The raw HGSC001-004 data are protected and are not available due to data privacy laws.
Data analysis	Methods section lists details on open source software that has been used together with Github link to the code base developed for this study. GitHub link: https://github.com/treangenlab/methphaser .

For manuscripts utilizing custom algorithms or software that are central to the research but not yet described in published literature, software must be made available to editors and reviewers. We strongly encourage code deposition in a community repository (e.g. GitHub). See the Nature Portfolio guidelines for submitting code & software for further information.

Data

Policy information about availability of data

All manuscripts must include a data availability statement. This statement should provide the following information, where applicable:

- Accession codes, unique identifiers, or web links for publicly available datasets
- A description of any restrictions on data availability
- For clinical datasets or third party data, please ensure that the statement adheres to our policy

Source data of Figure 2 for all available samples are stored at Supplementary Data 1. Source data of generating Figure 3 is stored at Supplementary Data 2. Source data for generating Figure 5 is stored at Supplementary Data 3 and 4. Source data for per-chromosome N50, switch error stats of HG002 with different read type

and coverages is stored at Supplementary Data 8-10.
The HG002 kit 10 R9 dataset is available at https://labs.epi2me.io/gm24385-5mc-remora/, called by Bonito base caller with profile dna_r9.4.1_e8_sup@v3.3. The reference genome is hg38 from Genome in a Bottle (GIAB NIST). The reads are at the coverage of 80x, and to test the effectiveness of our method in lower coverages, we also randomly subsampled the reads into 60x and 30x.
The HG002 kit 14 R10 dataset is available at https://humanpangenome.org/data.html. It is sequenced with Oxford Nanopore kit 14 (400bps speed) and pore version r10.4.1 and basecalled with Dorado v4.0.0 SUP model + Reomra. The reference genome is also hg38. The reads are at the coverage of 60X, and we also subsampled it into 30X.
Pangenome datasets' raw reads are available at https://github.com/human-pangenomics/hpgp-data. The raw reads are re-basecalled with Dorado + Remora the R9.4.1 data (SUP model, 5mCG modifications). The reference genome is also hg38. The samples' coverages are various, around 60X.
GIAB "high-confidence" region, also called Tier 1 region is provided by GIAB website, which is available at https://www.nist.gov/programs-projects/genome-bottle
All the HPRC cell-line VCF file and blood samples' (HGSC 001-004) block connection results from MethPhaser are available at Zenodo https://zenodo.org/doi/10.5281/zenodo.11195008. Please use the following template to provide all the information stated above:
The HGSC001-004 data generated in this study have been deposited in the Zenodo under accession code ZZ https://zenodo.org/doi/10.5281/zenodo.11195008. The raw HGSC001-004 data are protected and are not available due to data privacy laws.

Human research participants

Policy information about studies involving human research participants and Sex and Gender in Research.

Reporting on sex and gender
Use the terms sex (biological attribute) and gender (shaped by social and cultural circumstances) carefully in order to avoid confusing both terms. Indicate if findings apply to only one sex or gender; describe whether sex and gender were considered in study design whether sex and/or gender was determined based on self-reporting or assigned and methods used. Provide in the source data disaggregated sex and gender data where this information has been collected, and consent has been obtained for sharing of individual-level data; provide overall numbers in this Reporting Summary. Please state if this information has not been collected. Report sex- and gender-based analyses where performed, justify reasons for lack of sex- and gender-based analysis.

Population characteristics
Describe the covariate-relevant population characteristics of the human research participants (e.g. age, genotypic information, past and current diagnosis and treatment categories). If you filled out the behavioural & social sciences study design questions and have nothing to add here, write "See above."

Recruitment
Describe how participants were recruited. Outline any potential self-selection bias or other biases that may be present and how these are likely to impact results.

Ethics oversight
Identify the organization(s) that approved the study protocol.

Note that full information on the approval of the study protocol must also be provided in the manuscript.

Field-specific reporting

Please select the one below that is the best fit for your research. If you are not sure, read the appropriate sections before making your selection.

☒ Life sciences ☐ Behavioural & social sciences ☐ Ecological, evolutionary & environmental sciences

For a reference copy of the document with all sections, see nature.com/documents/nr-reporting-summary-flat.pdf

Life sciences study design

All studies must disclose on these points even when the disclosure is negative.

Sample size
9 individuals. The sample size is one flowcell of ONT sequencer, which is sufficient for clinical usage. Extra flowcell was applied on HG002 samples for more accurate results and comparisions.

Data exclusions
None

Replication
Replication can be performed by running the code with indicated parameters on the exact same data.

Randomization
The selection of public HPRC samples are evenly distributed by their ethnic backgrounds.

Blinding
No Blinding was applied because the HPRC data are public available and all the selected samples are used.

Reporting for specific materials, systems and methods

We require information from authors about some types of materials, experimental systems and methods used in many studies. Here, indicate whether each material, system or method listed is relevant to your study. If you are not sure if a list item applies to your research, read the appropriate section before selecting a response.

Materials & experimental systems

n/a	Involved in the study
☒	☐ Antibodies
☒	☐ Eukaryotic cell lines
☒	☐ Palaeontology and archaeology
☒	☐ Animals and other organisms
☒	☐ Clinical data
☒	☐ Dual use research of concern

Methods

n/a	Involved in the study
☒	☐ ChIP-seq
☒	☐ Flow cytometry
☒	☐ MRI-based neuroimaging
