# Supplementary material for: MethPhaser: methylation-based long-read haplotype phasing of human genomes
Source: Nat Commun. 2024 Jun 22;15:5327. doi: 10.1038/s41467-024-49588-0 (PMC11193733; doi:10.1038/s41467-024-49588-0)
Supplement: Supplementary file 3 — Description of Additional Supplementary Files [file 41467_2024_49588_MOESM3_ESM.pdf]

Description of Additional Supplementary Files

File Name: Supplementary Data 1

Description: Source Data of Figure 2, genome-wide stats of HG002

File Name: Supplementary Data 2

Description: Source Data of Figure 3, stats of per chromosome on R10 60X HG002

File Name: Supplementary Data 3

Description: Source Data of Figure 5a, genome-wide N50 stats of HPRC pangenome dataset

File Name: Supplementary Data 4

Description: Source Data of Figure 5b, genome-wide N50 stats of patient blood sample

File Name: Supplementary Data 5

Description: Comparison of with/without GIAB "high-confidence" region

File Name: Supplementary Data 6

Description: Comparison of different read length's impact on MethPhaser

File Name: Supplementary Data 7

Description: MethPhaser's Performance on HapCUT2 VS WhatsHap

File Name: Supplementary Data 8-10

Description: per-chromosme N50, switch error stats of HG002 with different read type and coverages

File Name: Supplementary Data 11

Description: The spanning information of phaseblocks on medically relevance genes on HG002 and pangenome samples

File Name: Supplementary Data 12

Description: Overall stats of the genome-wide phaseblocks spanning medically relecvance genes
